# Adhesions in the epidural space caused by frequent epidural blocks

**DOI:** 10.1186/s40981-017-0128-z

**Published:** 2017-10-11

**Authors:** Nobuhiro Shimada, Takashi Igarashi, Kunihiko Murai, Tetsuhito Hara, Tomoko Kuramochi, Mamoru Takeuchi

**Affiliations:** Department of Anesthesiology and Critical Care Medicine, Jichi Medical University, 3311-1, Yakushiji, Shimotsuke, Tochigi 329-0498 Japan

**Keywords:** Epidural block, Adhesions in the epidural space, Epiduroscopy

## Abstract

We report a case of adhesions in the epidural space caused by more than 200 times epidural blocks that were observed with epiduroscopy. A 41-year-old man had repeatedly undergone lumbar epidural blocks to treat pain in his leg, resulting in decreased efficacy of the epidural block. We described endoscopic findings that these adhesions were mostly consisted of adhesions formed from the soft connective tissue.

## Background

Repeated long-term single or continuous epidural blocks are considered to cause adhesions in the epidural space, resulting in decreased effects of the epidural block. Few reports have detailed epidural space anatomical findings and treatment for such cases [1]. We report our observations of the epidural space in a patient who underwent frequent epidural blocks.

## Case presentation

A 41-year-old man with no significant medical history presented with a chief complaint of pain in his left leg. The top of his left foot had been run over by a car tire. No fractures or joint injuries were noted in the toes. He was diagnosed with complex regional pain syndrome (CRPS).

He underwent twice-weekly lumbar epidural blocks at his local hospital and a month-long continuous epidural block each winter, which allowed him to return to work. The needle used for a single epidural block was 20 gauge, and the catheter used for the continuous epidural block was 20 gauge. The degree of pain in his left leg according to the numerical rating scale (NRS) was 8 during activities of daily living and 3 after a lumbar epidural block was performed.

Five years after the initiation of epidural blocks, greater resistance was felt while injecting the medication into the epidural space, and it was difficult to insert the catheter. The degree of pain in the leg after the epidural block became NRS 6, indicating that effects of the epidural block were insufficient. The degree of pain in the leg during activities of daily living was not exacerbated, remaining at NRS 8. Adhesions in the epidural space caused by frequent epidural blocks were suspected, and the patient was referred to our department.

**Fig. 1:**
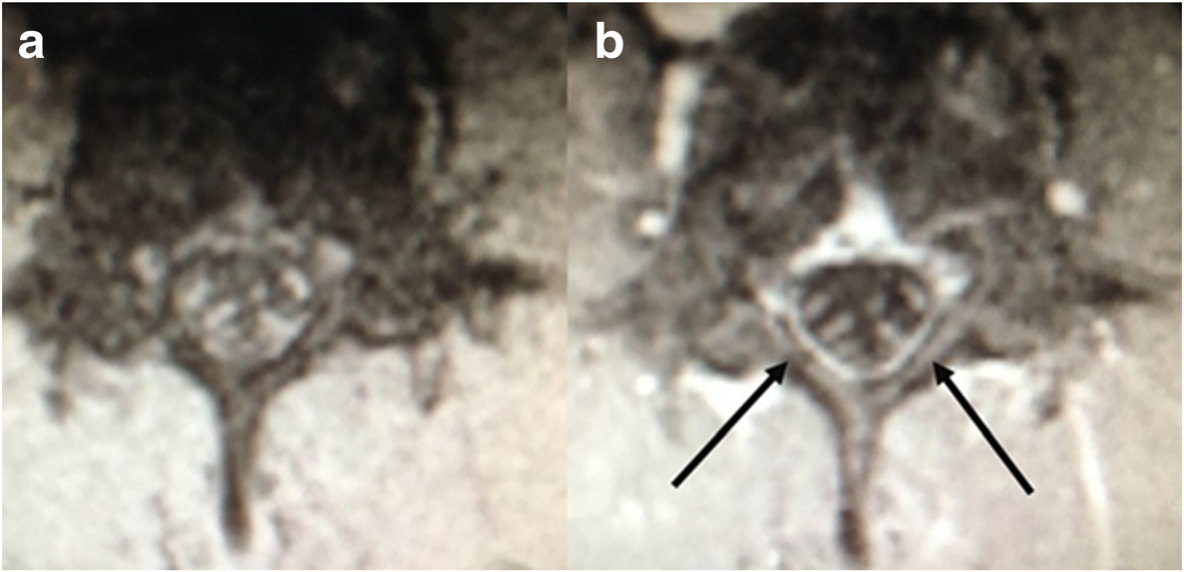
MRI. Transverse section of the L4 vertebral area. **a** Unenhanced fat-suppressed T1-weighted image. **b** Gd-contrast enhanced fat-suppressed T1-weighted image. Arrow indicates contrast effects observed in the back of the epidural space

The patient had undergone epidural blocks many times at his local hospital. These comprised single epidural blocks with 10 ml 0.375–0.75% ropivacaine at the L4/5 and L5/S1 levels performed over 200 times as well as continuous epidural blocks using 0.2% ropivacaine for approximately 1 month performed multiple times. During examination, the patient exhibited spontaneous pain from the left leg to the foot accompanied by allodynia. Muscular atrophy and edema were also noted at the same site. Manual muscle testing results ranged from 2 to 3 in the tibialis anterior muscle, triceps surae muscle, gastrocnemius muscle, long extensor muscle of the thumb, and flexor hallucis longus muscle. There was no decreased perception and no other abnormal neurological findings. In the area from the left leg to the foot, plain X-rays revealed mild bone atrophy and magnetic resonance imaging (MRI) revealed mild bone and muscle atrophy. No abnormalities were noted in the lumbar spine on plain X-rays, and MRI did not reveal any abnormal findings in the intervertebral discs, vertebrae, spine, or nerve roots. T1-weighted fat-suppressed images showed uneven contrast effects of gadolinium throughout the back of the epidural space from L4 to S1 (Fig. 1). Epidurography performed from the sacral hiatus revealed blockage in the L5/S1 vertebral area. Lower limb thermography revealed decreased temperature throughout the entire left leg. We diagnosed the pain in his left foot as CRPS because it applied to continuing pain which was disproportionate to any inciting event, hyperesthesia, allodynia, temperature asymmetry, motor dysfunction, and trophic changes among the diagnostic criteria for CRPS by International Association for the Study of Pain, 2005 [2]. However, the attenuated effects of the epidural block were determined to be due to adhesions in the epidural space rather than CRPS exacerbation.

Regarding further treatment, the patient was advised that, although it might be possible to regain the effect of the epidural block by performing adhesiotomy in the epidural space, this was not a radical cure for CRPS of the left leg, and it would be difficult to achieve good-quality continuous epidural blocks over long periods if adhesions in the epidural space recurred following adhesiotomy. Therefore, he was advised to undergo spinal cord stimulation rather than long-term epidural blocks as a means of treating the pain in his leg. However, the patient requested to undergo adhesiotomy in the epidural space.

**Fig. 2:**
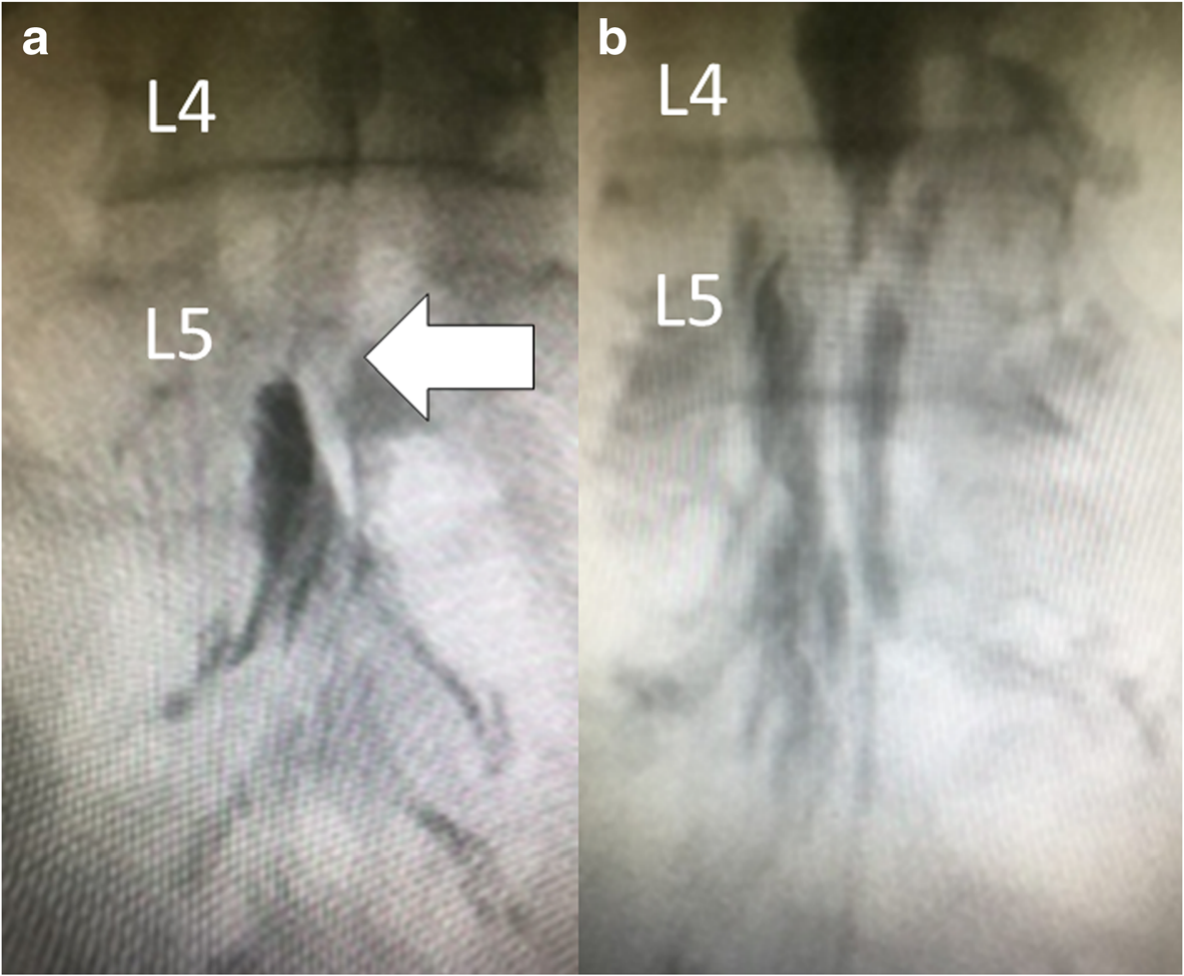
Epidurography. **a** Before epiduroscopy, there was blockage of the L5/S1 vertebral area (arrow), with no contrast of the epidural space any further toward the cranial side of this area. **b** After epiduroscopy, contrast effects were achieved the cranial side of the epidural space from the L5 vertebral area

**Fig. 3:**
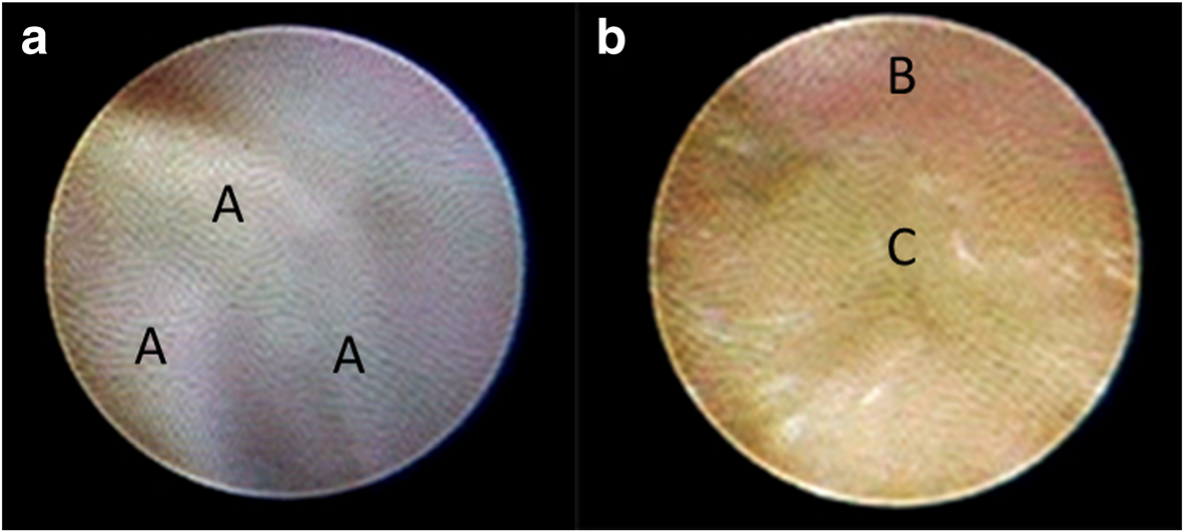
Endoscopic findings in the epidural space. **a** There were adhesions in the L5 vertebral area. Due to the connective tissue, we could not get the clear view. There were increases in the fibrous tissue in the part of the connective tissue (A). These adhesions were mostly formed from the soft connective tissue. **b** In the L3 vertebral area, dura mater (B) and fatty tissue (C) could be clearly observed and appeared to be normal

Therefore, epiduroscopy was performed. We used video-guided catheter and 0.9 mm fiberopticscope (Kobamed system, Japan Medicalnext Co., Ltd., Osaka, Japan). Contrast enhancement of the area from the sacral hiatus to the epidural space performed before epiduroscopy revealed blockage of the L5/S1 vertebral area, with no contrast of the epidural space any further toward the cranial side of this area (Fig. 2a). Endoscopic findings revealed adhesions in the epidural space in the L4 to S1 vertebral area. The adhesions were mainly formed from the soft connective tissue, and little hard scar tissue was observed. The adhesions were distributed throughout the L4 to S1 vertebral area, with many adhesions observed on the left side of the L4 and L5 vertebral area. There were few areas of hyperemia, reddening, or blood vessel proliferation (Fig. 3a). Adhesiotomy was carefully performed using the catheter tip while injecting the physiological saline solution. We were able to easily detach the adhesions, and contrast effects were achieved in the epidural space (Fig. 2b). In the epidural space toward the cranial side from the L3 vertebral area, dura mater and fatty tissue could be clearly observed and appeared to be normal (Fig. 3b). The operation time was 58 min, and the physiological saline solution used for adhesiotomy was 250 ml.

After epiduroscopy, although the degree of pain in his leg after an outpatient epidural block had recovered to NRS 4, the effects of once-weekly epidural blocks were only sufficient for 1 month. At this point, the patient realized that the treatment of the pain in his leg with epidural blocks would be difficult and requested to undergo spinal cord stimulation.

For spinal cord stimulation, an 8-pole electrode (Octrode™, Medtronic, Inc., Minneapolis, MN, USA) was percutaneously placed into the epidural space so that the tip was in the Th9 vertebral area and a nerve stimulator (Eon Mini™, Medtronic, Inc) was placed in the right hypogastric region. Thereafter, the degree of pain in his leg during activities of daily living decreased to NRS 5.

### Discussion

In our patient, the effects of repeated epidural blocks gradually decreased. MRI, epidurography, and epiduroscopy indicated adhesions at epidural block puncture sites. Furthermore, no spinal disorders were observed on imaging. Therefore, our patient appeared to have adhesions caused by repeated epidural blocks.

MRI and epidurography are generally used to diagnose adhesions in the epidural space. When adhesions are present in the epidural space, MRI shows enhanced contrast effects in the connective tissue in the epidural space and disappearance of epidural fat [3, 4]. Although MRI is noninvasive and can be used for extensive observation of adhesions, its diagnostic precision is low. Epidurography shows blockages at the adhesion sites. It can be used for extensive observation of adhesions and offers high diagnostic precision. However, the diagnosis of adhesions located further toward the epidural space on the cranial side from the blockage requires another puncture site. Although MRI and epidurography are sufficient for the diagnosis of epidural adhesion, it may be possible to obtain findings of epidural space by epiduroscopy which are difficult to obtain by other radiological tests.

Epidural blocks result in connective tissue growth in the epidural space due to microbleeding caused by the punctures, local inflammation caused by high concentration of local anesthetics, and healing process of the inflammation [5]. In our patient, it appeared that multiple epidural blocks and continuous epidural catheter insertion have contributed to this connective tissue growth. Most of the adhesions in our patient resulted from connective tissue growth, and there were few areas with strong adhesions, scarring, hyperemia, or reddening. In contrast, standard endoscopic findings of lumbar degenerative diseases such as lumbar disc herniation, lumbar spinal canal stenosis, and failed back surgery syndrome have been reported [6, 7]. In case of lumbar disc herniation, inflammatory findings such as hyperemia and reddening are common and adhesions are often mild. In case of lumbar spinal canal stenosis, inflammatory findings and connective tissue growth are observed due to the degree of stenosis, and adhesions are often strong. In cases of failed back surgery syndrome, the connective tissue in the epidural space is known to form scars and exhibit strong adhesions around the nerves. Comparing our endoscopic findings to previous reports, the connective tissue that developed in our patient because of epidural blocks was milder than any of other lumbar degenerative diseases.

Normally, lumbar and sciatic nerve pain due to lumbar herniated disc, lumbar canal stenosis, and failed back syndrome are indications for epiduroscopy [7]. We performed adhesiotomy in the epidural space in our patient to enable him to undergo subsequent epidural blocks as treatment. Although adhesions in the epidural space could be detached with epiduroscopy, adhesions recurred because of repeated epidural blocks following adhesiotomy. Therefore, it appears that performing adhesiotomy and subsequent epidural blocks cannot be recommended for such cases of iatrogenic adhesions of epidural space even when the patient asks to perform.

## Conclusions

We described the epiduroscopy findings in the epidural space in which adhesions had formed because of frequent epidural blocks. Adhesions in the epidural space were noted at the epidural block puncture sites. Most adhesions involved the growth of the soft connective tissue, and little hard scar tissue was observed.

## Abbreviations

CRPS: Complex regional pain syndrome
MRI: Magnetic resonance imaging
NRS: Numerical rating scale
